# Evaluating Platelet-to-Lymphocyte Ratio and Systemic Immune-Inflammation Index as Distinctive Biomarkers in Type 2 Diabetes Mellitus Patients With and Without Proteinuria: A Retrospective Study

**DOI:** 10.7759/cureus.79348

**Published:** 2025-02-20

**Authors:** Shubhransu Patro, Arushi Choudhary, Vibha Sharma, Apoorav Mahajan, Diptiman Sahoo, Sidharth S Pattnaik

**Affiliations:** 1 General Medicine, Kalinga Institute of Medical Sciences, Bhubaneswar, IND; 2 Internal Medicine, S.C.B. Medical College and Hospital, Cuttack, IND

**Keywords:** biomarkers, chronic inflammation, diabetic kidney disease, hba1c, platelet-to-lymphocyte ratio, proteinuria, systemic inflammatory index, type 2 diabetes mellitus, urine acr

## Abstract

Background

Diabetic kidney disease (DKD), a leading cause of chronic kidney disease (CKD) and end-stage renal disease globally, is driven by metabolic and inflammatory processes. Systemic immune-inflammation index (SII) and platelet-to-lymphocyte ratio (PLR) are emerging biomarkers that integrate inflammatory and hematological components, potentially reflecting severity of renal dysfunction.

Methods

This retrospective study analyzed 160 patients from January 2023 to December 2023 with type 2 diabetes mellitus (T2DM), equally divided into proteinuria and non-proteinuria groups. SII and PLR levels were compared between groups, and their correlations with urine albumin-to-creatinine ratio (ACR) and protein levels were evaluated using statistical measures, including Pearson’s correlation and regression analyses.

Results

SII and PLR levels were significantly higher in the proteinuria group (SII: 1,305,266 vs. 456,957.4 cells per microliter; PLR: 252.09 vs. 99.74, p<0.001 for both). SII and PLR exhibited strong positive correlations with urine ACR (SII: r=0.8537, PLR: r=0.8362) and protein levels (SII: r=0.834, PLR: r=0.841, p<0.01 for both). Regression analysis revealed a complex, nonlinear association between these biomarkers and increasing proteinuria severity, indicating their dynamic behavior across protein categories. HbA1c demonstrated a parabolic relationship with urine ACR, with progressively larger effects at higher levels, highlighting its role in worsening renal dysfunction.

Conclusion

SII and PLR are robust markers of inflammation and renal injury in T2DM, showing strong associations with proteinuria and urine ACR. These indices, alongside HbA1c, may aid in early detection and monitoring of DKD progression, particularly in resource-constrained settings.

## Introduction

Diabetes mellitus (DM) has emerged as a global epidemic, affecting approximately 11% of the global population in 2021, with projections indicating an increase to 12% by 2045 [1,2]. While type 2 diabetes mellitus (T2DM) accounts for nearly 90% of this burden and poses significant risks for developing complications, including diabetic kidney disease (DKD), an estimated 30-40% of individuals with T2DM also progress to DKD during their lifetime [3]. Although it took three millennia to find an association between DM and renal disease, DKD is now recorded as the leading cause of chronic kidney disease (CKD) and end-stage kidney disease, contributing to nearly half of all cases worldwide [4]. The impact of DKD is substantial, with kidney disease ranking as the 12th most common cause of mortality globally, accounting for approximately 1.1 million deaths annually and showing a 31.7% increase in mortality over the past decade [2-3]. The pathogenesis of DKD is an interplay of metabolic and hemodynamic factors driven by hyperglycemia and insulin resistance (IR). These conditions create a pro-inflammatory milieu, exacerbating renal damage. IR has been strongly linked to chronic low-grade inflammation, mediated by cytokines such as interleukin-1 (IL-1), interleukin-6 (IL-6), and tumor necrosis factor-alpha (TNF-α). Consequently, DKD is now considered as immunological disorder, manifesting both systemic and local inflammatory responses at the renal level [5].

While established markers such as TNF-alpha and high-sensitivity C-reactive protein (hsCRP) provide valuable insights into the inflammatory processes underlying DKD, these biomarkers are costly and require advanced laboratory setups, making them less feasible in resource-constrained settings [5]. Therefore, there is an urgent need for readily available markers that can aid in the early detection and management of DKD, especially in its subclinical stages where renal injury precedes albumin excretion.

Currently, DKD is diagnosed based on urine albumin-to-creatinine ratio ([ACR] ≥ 30 mg/g) and/or reduced estimated glomerular filtration rate (eGFR <60 mL/min/1.73 m²) for more than three months. However, these parameters have limitations in detecting early renal damage, as subclinical injury often occurs before significant albuminuria [3,5]. Therefore, exploring alternative biomarkers is crucial and need of the hour.

Various studies reflect systemic inflammation and its role in DKD progression. Emerging evidence suggests that hematological indices, such as the systemic immune-inflammation index (SII) and platelet-to-lymphocyte ratio (PLR), may serve as promising indicators of inflammation and vascular dysfunction in DKD. SII, calculated as (platelet count × neutrophil count) / lymphocyte count, provides a composite measure of immune and inflammatory status, incorporating three critical components of inflammation. Initially developed to assess prognosis in solid tumors, it has shown promising potential in evaluating inflammatory and thrombotic processes in other diseases, including DKD. Similarly, PLR has been proposed as a surrogate marker of endothelial dysfunction and chronic inflammation, making it a valuable tool for identifying vascular complications in T2DM [6-8].

The aim of the study is to correlate the role of PLR and SII in differentiating T2DM patients with and without proteinuria. Given the inflammatory basis of DKD and its strong association with glycemic control, inflammatory indices such as SII and PLR could provide valuable insights into disease progression. By integrating these markers into clinical practice, it may be possible to enhance the early detection of DKD, monitor disease activity, and improve patient outcomes in resource-limited settings.

## Materials and methods

Study design and duration

This retrospective study was conducted over a one-year period from January 2023 to December 2023 in the Department of General Medicine, Pradyumna Bal Memorial Hospital, KIMS, Kalinga Institute of Medical Sciences, Bhubaneswar, Odisha, India. The study aimed to evaluate the role of the PLR and SII in differentiating patients with T2DM with and without proteinuria.

Inclusion and exclusion criteria

The study included adult patients diagnosed with T2DM as per the American Diabetes Association (ADA) criteria 2025 [9]. Patients were excluded if they had active inflammatory or infectious diseases, malignant conditions, hematological malignancies, were pregnant, under the age of 18 years, diagnosed with type 1 diabetes mellitus, or using medications affecting platelet counts.

Study population and grouping

Eligible patients were grouped into two categories based on urine ACR levels. Those with elevated ACR levels (>30 mg/g) were categorized as the proteinuria group, while patients with normal ACR levels (≤30 mg/g) were categorized as the non-proteinuria group. Consecutive sampling was employed to recruit participants during the study period.

Data collection

Data were collected through patient interviews, clinical examinations, and laboratory tests. Demographic details, including name, age, and sex, were recorded alongside clinical profiles, which included medical history, present illness, antibiotic history, and past and personal history. Comprehensive general and systemic clinical examinations were conducted.

Laboratory investigations

All patients underwent routine laboratory investigations, including a complete blood count to analyze inflammatory and hematological markers, HbA1c levels to assess glycemic control, serum urea and creatinine to evaluate renal function, urine ACR to classify patients into proteinuria or non-proteinuria groups, and urine routine microscopy (R/M) to identify any associated urinary abnormalities.

The eGFR was calculated using the Chronic Kidney Disease Epidemiology Collaboration (CKD-EPI) algorithm to further assess renal function.

Statistical analysis

Data analysis was performed using R (version 4.3.2) programming software (R Foundation for Statistical Computing, Vienna, Austria) [10]. Continuous variables with normal distribution were compared between groups using the independent samples t-test, and results were expressed as mean ± standard deviation (SD). For non-normally distributed variables, the Mann-Whitney U test was employed, and results were expressed as median (range). Categorical variables were analyzed using the chi-square test.

Correlation between study parameters, including PLR, SII, and clinical measures, was assessed using Pearson’s correlation analysis. Multinomial and polynomial logistic regression models were used to further explore the relationship between predictors and the outcomes. A p-value of <0.05 was considered statistically significant. Results were reported in terms of mean differences, correlation coefficients, and p-values to elucidate significant findings comprehensively.

Ethical approval and consent

This study was performed in accordance with the principles of the Declaration of Helsinki, with ethical approval obtained from the Institutional Ethics Committee of Kalinga Institute of Medical Sciences (KIIT/KIMS/IEC/1751/2024) prior to the study's commencement. Patient confidentiality was rigorously maintained, and data were anonymized during analysis to protect patient privacy.

## Results

Demography

A total of 160 patients were analyzed, divided equally into two groups: 80 with diabetic proteinuria and 80 with DM but no proteinuria (Table 1). Among the proteinuria group, 53 (66.25%) were male, while in the non-proteinuria group, 54 (67.5%) were male. The mean hemoglobin (Hb) level in the proteinuria group was 9.97 g/dL compared to 10.44 g/dL in the non-proteinuria group. In terms of comorbidities in the proteinuria group, 47 patients (58.75%) had hypertension (HTN), 14 (17.5%) had chronic renal failure (CRF), 14 (17.5%) had cardiovascular disease (CVD), 26 (32.5%) had anemia, 3 (3.75%) had pulmonary HTN, and 17 (21.25%) had other conditions. Conversely, in the non-proteinuria group, 30 patients (37.5%) had HTN, 2 (2.5%) had foot ulcers, 12 (15%) had CRF, 12 (15%) had CVD, 2 (2.5%) had chronic liver disease, 8 (10%) had peripheral vascular disease, and 19 (23.75%) had other conditions.

**Table 1:** Demographic distribution The demographic data have been expressed as frequency (proportion).

Group	Proteinuria (n=80)	Non-proteinuria (n=80)
Gender		
Male	53 (66.25%)	54 (67.50%)
Female	27 (33.75%)	26 (32.50%)
Hemoglobin	9.97 (mean)	10.44 (mean)
Comorbidities		
Hypertension	47 (58.75%)	30 (37.50%)
Chronic renal failure	14 (17.50%)	12 (15.00%)
Cardiovascular disease	14 (17.50%)	12 (15.00%)
Anemia	26 (32.50%)	-
Pulmonary hypertension	3 (3.75%)	-
Foot ulcer	-	2 (2.50%)
Chronic liver disease	-	2 (2.50%)
Peripheral vascular disease	-	8 (10.00%)
Other comorbidities	17 (21.25%)	19 (23.75%)

Comparative analysis of SII and PLR

The analysis revealed significant differences in SII and PLR between patients with and without proteinuria. The mean SII was markedly higher in patients with protein in their urine, measuring 1,305.266 cells per microliter compared to 456,957.4 cells per microliter in those without proteinuria. This difference was statistically significant, as indicated by a T-statistic of 8.9101 and a p-value of less than 0.001. The 95% confidence interval for the mean difference ranged from 658,037.4 to 1,038,580.0, underscoring the reliability of the observed disparity. Similarly, the mean PLR was substantially elevated in patients with proteinuria, with a value of 252.09 compared to 99.74 in those without. This difference was also highly significant, with a T-statistic of 10.845 and a p-value below 0.001. The 95% confidence interval for the difference in PLR ranged from 124.31 to 180.40, further supporting the robustness of this finding.

**Figure 1:**
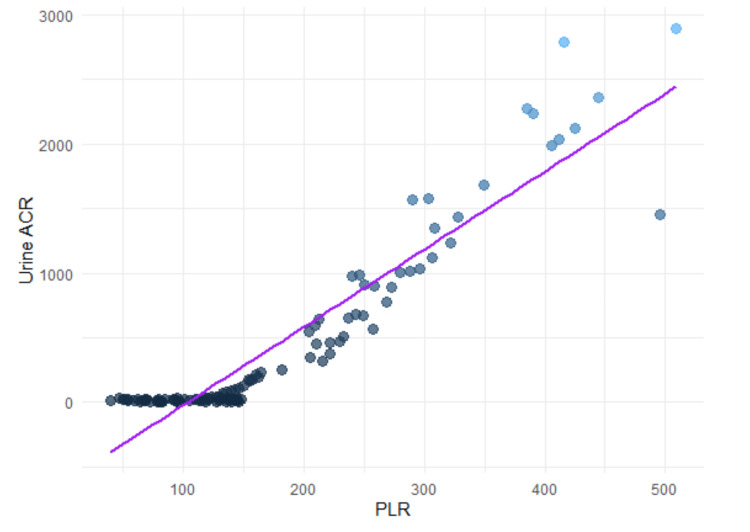
Scatterplot of PLR versus urine ACR ACR, albumin-to-creatinine ratio; PLR, platelet-to-lymphocyte ratio

**Figure 2:**
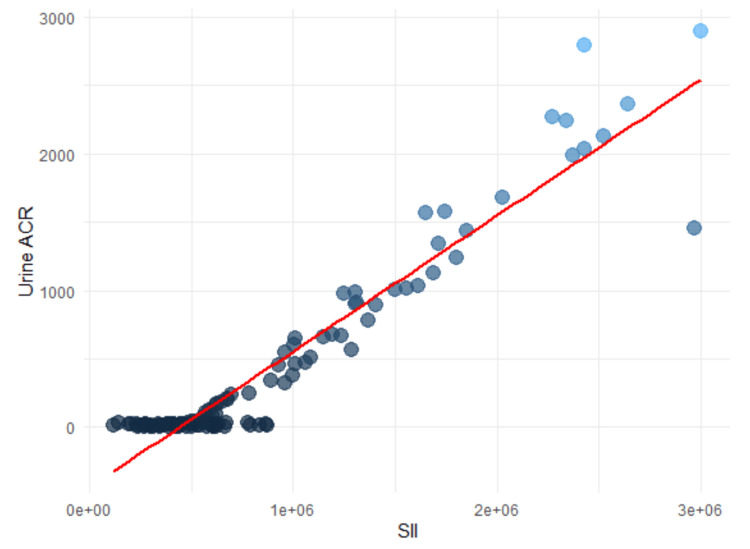
Scatterplot of SII versus urine ACR ACR, albumin-to-creatinine ratio; SII, systemic immune-inflammation index

Additionally, both SII and PLR exhibited strong positive correlations with the urine ACR, suggesting their potential utility as biomarkers for assessing proteinuria-related conditions. The correlation coefficient for SII and urine ACR was 0.8537, with a 95% confidence interval of 0.8333 to 0.8680, indicating a highly consistent association across the dataset. PLR also demonstrated a strong correlation with urine ACR, with a correlation coefficient of 0.8362 and a 95% confidence interval ranging from 0.8085 to 0.8557. These findings highlight the clinical relevance of SII and PLR as indicators of inflammation and renal dysfunction in patients with proteinuria (Figures 1, 2).

Correlation analysis (SII and PLR versus urine protein)

The correlation analysis demonstrated a very strong positive relationship between both the SII and PLR with urine protein levels. SII showed a Pearson correlation coefficient of 0.834, with a p-value of less than 0.01 and a 95% confidence interval ranging from 0.8058 to 0.8544. This indicates a highly significant and consistent association, with SII values progressively increasing as urine protein levels rise, from a mean of 456,957 in the "absent" category to 2,328,593 in the "4+" category. Similarly, PLR exhibited a Pearson correlation coefficient of 0.841, also with a p-value below 0.01 and a confidence interval of 0.8148 to 0.8589. The mean PLR also increased steadily across protein categories, from 99.7 in the "absent" group to 396.0 in the "4+" group. These findings strongly support the utility of both SII and PLR as reliable biomarkers for increasing levels of proteinuria.

**Figure 3:**
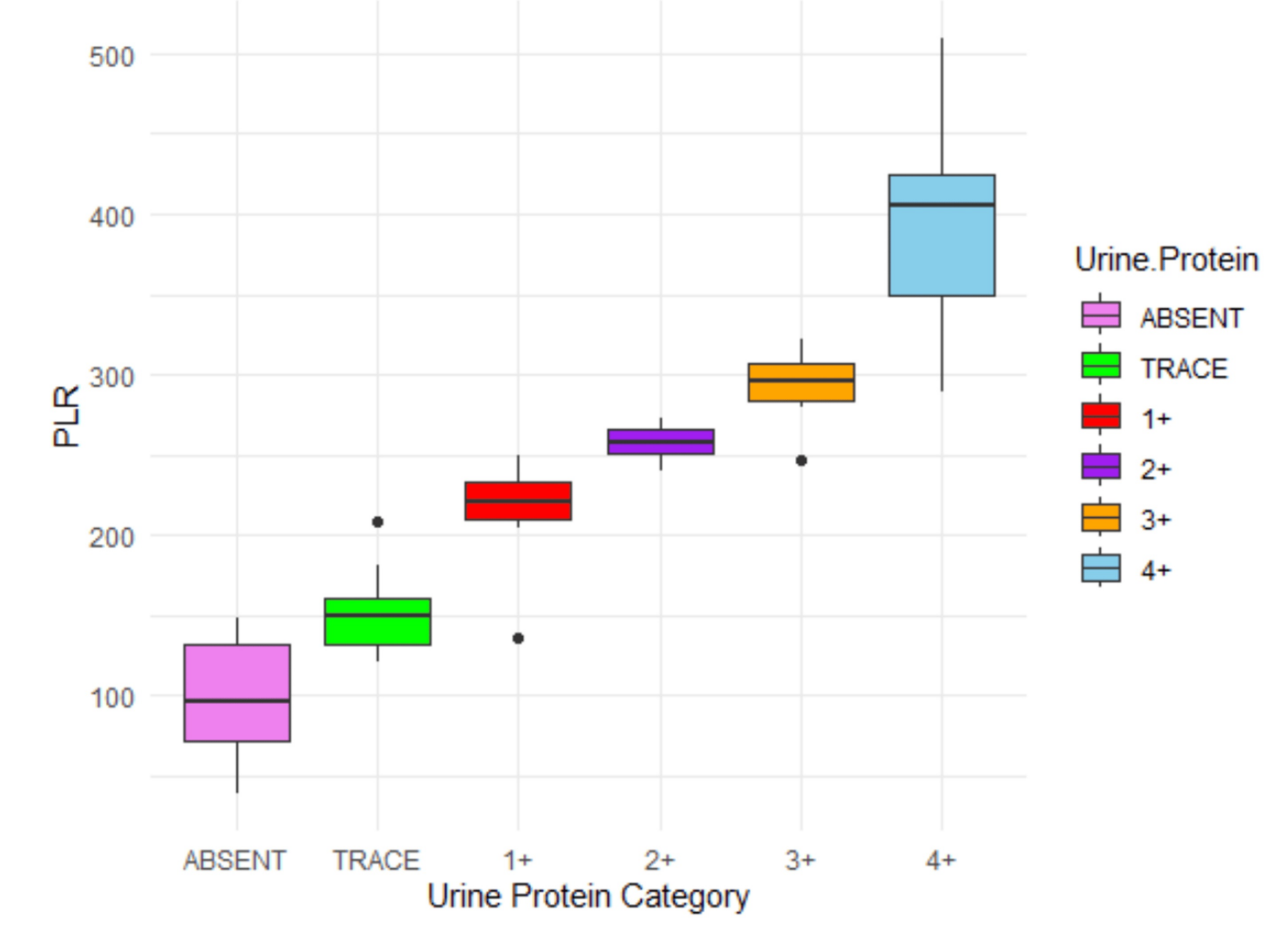
Boxplot of distribution of PLR in different proteinuria categories PLR, platelet-to-lymphocyte ratio

**Figure 4:**
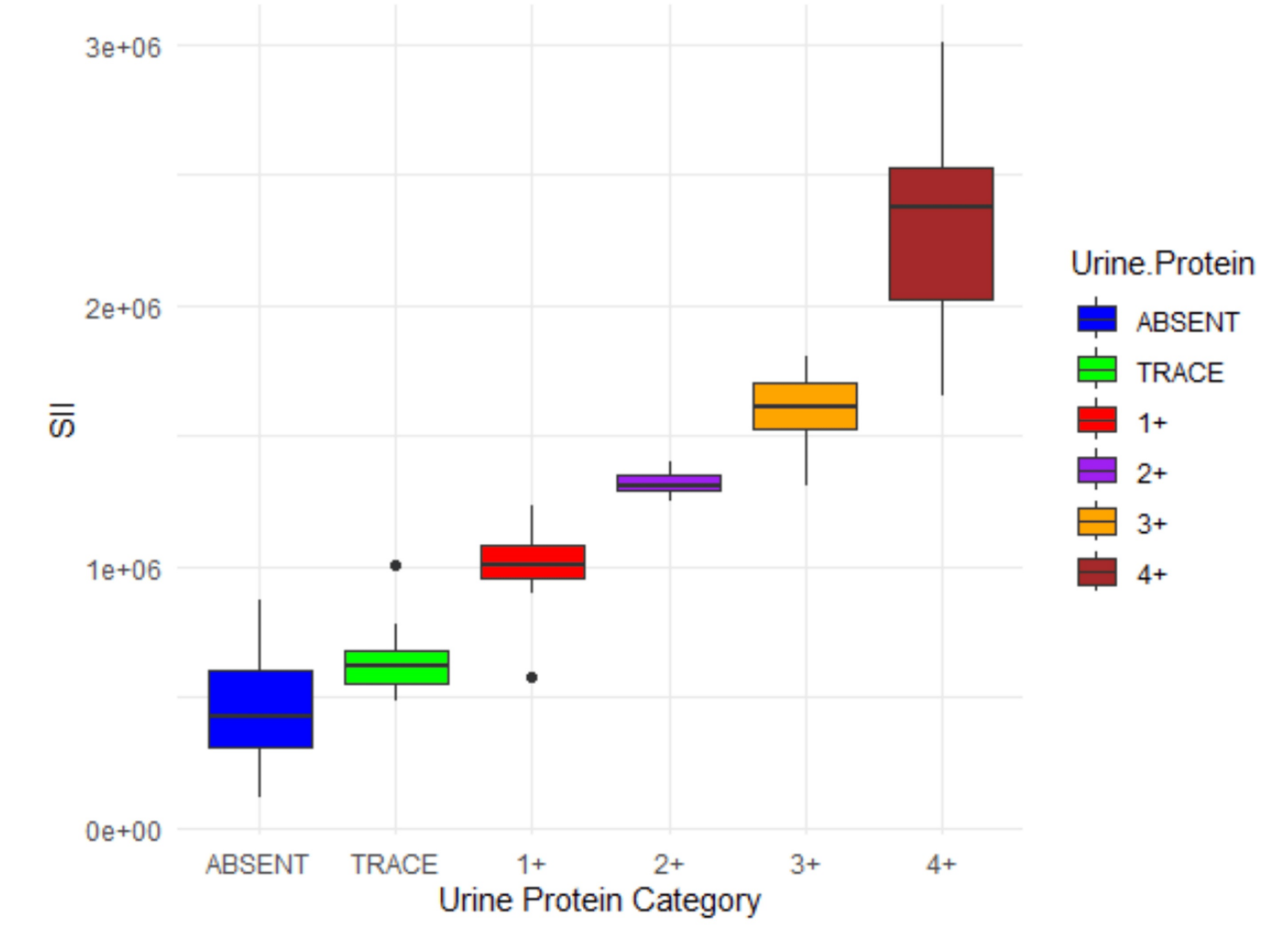
Box plot of distribution of SII in different proteinuria categories SII, systemic immune-inflammation index

The analysis reveals distinct insights into the relationship between PLR, SII, and urine protein levels through correlation and multinomial logistic regression (Table 2). The correlation analysis showed a strong, positive linear relationship between PLR and urine protein levels (correlation coefficient = 0.84), indicating that PLR generally increases with higher protein levels (Figure 3). However, multinomial logistic regression, which models the probability of urine protein categories (e.g., trace, 1+, 2+) relative to the baseline (absent), suggests a more complex, nonlinear interaction. While SII consistently showed positive coefficients across categories except for trace (Figure 4), PLR exhibited negative coefficients at higher protein levels (3+, 4+), reflecting a nuanced relationship. This suggests that PLR initially increases with lower protein categories in a linear fashion but interacts differently with higher levels, potentially due to thresholds or nonlinear effects in the data.

**Table 2:** Regression analysis of the predictors and urine protein The p-values are for multinomial regression analysis PLR, platelet-to-lymphocyte ratio; SII, systemic immune-inflammation index

Urine protein category	Intercept (log-odds)	SII coefficient	PLR coefficient	P-value for SII	P-value for PLR
Trace	-11.528	-3.82	0.081	0.524	0.48
1+	24.952	1.18	0.146	0.224	0.01
2+	123.764	1.39	0.140	0.01	0.01
3+	288.368	6.57	-2.221	0.01	0.052
4+	478.39	1.50	-6.382	0.049	0.051

HbA1c versus urine ACR and urine protein

**Figure 5:**
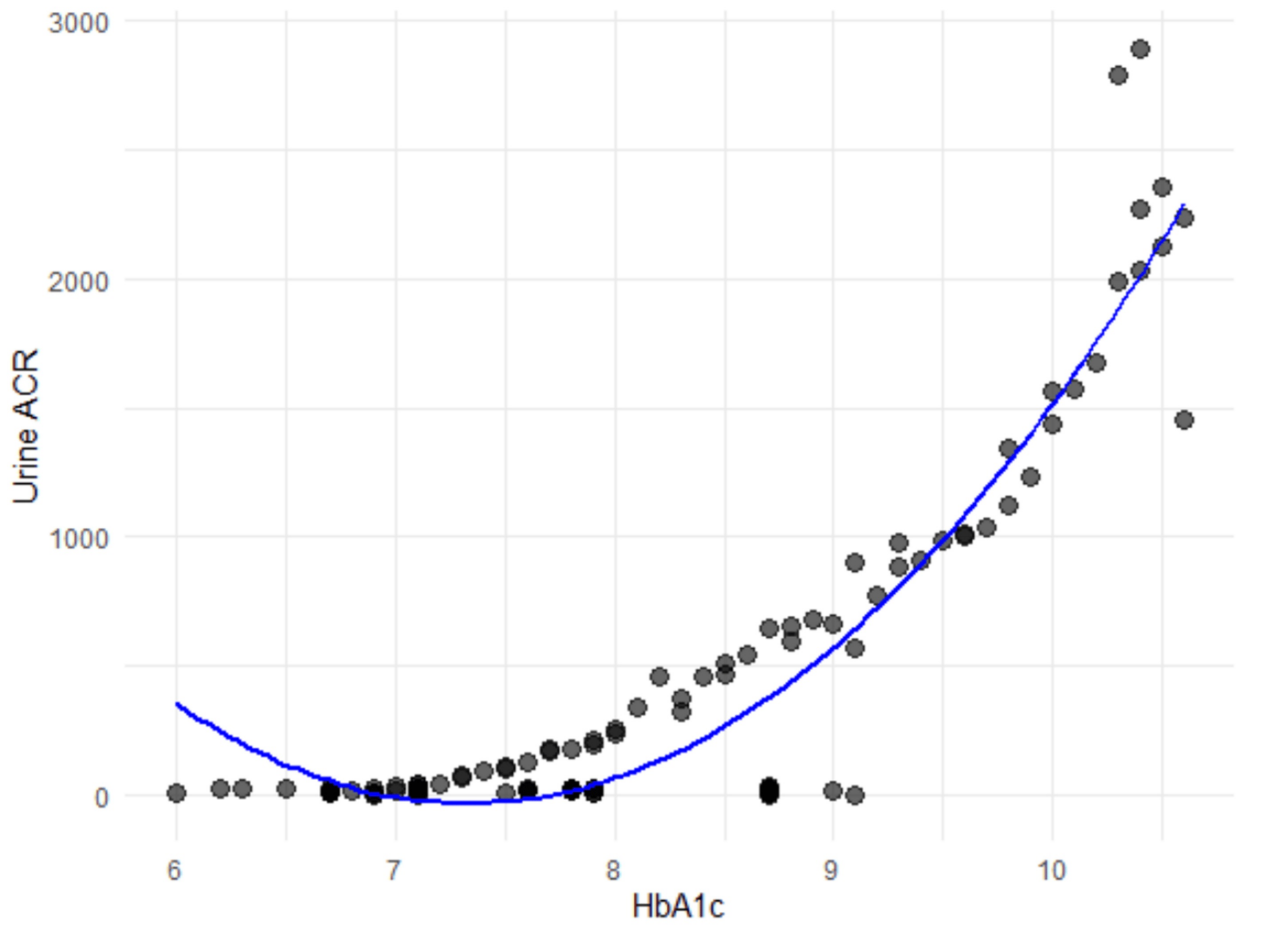
Polynomial regression between HbA1C and urine ACR ACR, albumin-to-creatinine ratio

The relationship between HbA1c and urine ACR shows a strong positive association, with urine ACR increasing by an average of 482.73 mg/g for every unit rise in HbA1c. This linear model explains about 68.12% of the variation in urine ACR (R² = 0.6812), and the residual standard error of 386.5 indicates the typical deviation of observed values from predictions. A more detailed quadratic model reveals a curved, parabolic relationship: while urine ACR rises steadily with increasing HbA1c (linear coefficient = 5964.7), it accelerates at higher HbA1c levels due to the significant quadratic term (coefficient = 3215.7) (Figure 5). This pattern suggests that as HbA1c becomes higher, the impact on urine ACR grows disproportionately, potentially reflecting worsening kidney function at elevated HbA1c levels.

The analysis of HbA1c as a predictor of urine protein levels revealed varying degrees of association across protein categories compared to the absent category (Table 3). For the trace category, HbA1c did not show a significant association, indicating no meaningful link between HbA1c and trace protein levels. However, significant positive associations were observed for HbA1c with protein levels in the 1+, 2+, 3+, and 4+ categories. The strength of association increased progressively with higher protein categories, with HbA1c coefficients of 1.555 for 1+, 9.046 for 2+, 16.444 for 3+, and 26.257 for 4+, all with highly significant p-values (<0.01). These results suggest that higher HbA1c levels are strongly associated with greater proteinuria severity, highlighting its potential role as a predictor for advancing proteinuria.

**Table 3:** Correlation analysis between HbA1c and urine protein levels

Category	Term	Coefficient	Standard error	Z - score	P-value for Pearson correlation	Interpretation
Trace	HbA1c	0.067	0.377	0.179	0.8583	HbA1c does not significantly predict trace protein level.
1+	HbA1c	1.555	0.509	3.053	0.0023	A significant positive association between HbA1c and 1+ protein level.
2+	HbA1c	9.046	3.237	2.796	0.0052	A significant positive association between HbA1c and 2+ protein level.
3+	HbA1c	16.443	5.198	3.164	0.0016	A significant positive association between HbA1c and 3+ protein level.
4+	HbA1c	26.2566	7.088	3.704	0.0002	A significant positive association between HbA1c and 4+ protein level.

## Discussion

The findings from this study illuminate the intricate interplay between systemic inflammation, glycemic control, and renal dysfunction in DM, with SII and PLR emerging as promising biomarkers for assessing proteinuria-related conditions. Elevated levels of SII and PLR observed in patients with diabetic proteinuria highlight the pervasive inflammatory milieu in these individuals, underscoring the pathogenic role of inflammation in the progression of renal damage [8]. These results resonate with existing evidence in the literature, where inflammation is increasingly recognized as a critical driver of microvascular complications, including diabetic nephropathy [11]. The significantly higher SII values in the proteinuria group, as compared to those without proteinuria, strongly suggest that this index is a robust indicator of systemic inflammation linked to renal impairment [8,12]. SII, which integrates neutrophil, platelet, and lymphocyte counts, reflects a heightened inflammatory and prothrombotic state. Such inflammatory cascades, exacerbated by chronic hyperglycemia, contribute to endothelial dysfunction and glomerular injury, pivotal events in the pathogenesis of proteinuria [7,13].

Previous studies have substantiated these findings, with Yan et al. demonstrating that elevated SII is independently associated with the severity of diabetic nephropathy and its progression to end-stage renal disease. They evaluated 1,922 patients with T2DM, revealing a significant positive association between higher SII levels and increased DKD prevalence and severity, as measured by urinary ACR and eGFR. Logistic regression showed that SII independently predicted DKD presence, with higher SII quartiles correlating with greater odds of DKD [14]. Similarly, other research has highlighted the predictive value of SII in cardiovascular and renal outcomes among diabetic populations, reinforcing its clinical relevance as a biomarker [15].

The parallel elevation in PLR among proteinuria patients provides additional insights into the inflammatory processes underpinning renal dysfunction [16]. PLR, a simpler yet effective inflammatory marker, balances the pro-inflammatory activity of platelets against the regulatory roles of lymphocytes. Its association with proteinuria severity observed in this study aligns with findings from Li et al., who reported a strong link between elevated PLR and microvascular complications, including nephropathy, retinopathy, and neuropathy, in diabetic patients. Such correlations emphasize the importance of these indices not only in detecting inflammation but also in quantifying its impact on renal function [17]. These findings simulate our results in terms of exploring SII and PLR as biomarkers of proteinuria and progressive renal damage.

The correlations of both SII and PLR with the urine ACR underscore their potential as surrogate markers of renal injury [18]. In this study, the strong positive relationships observed highlight that as proteinuria severity increases, so do the levels of SII and PLR, reflecting escalating systemic inflammation. This dynamic relationship supports their clinical utility in stratifying risk and monitoring disease progression in DM-related kidney disease. Trimarchi et al. similarly identified robust associations between inflammatory biomarkers and urinary protein excretion, further validating these findings [19].

The relationship between glycemic control, as indicated by HbA1C, and proteinuria severity adds another dimension to understanding the mechanisms of renal injury [20]. In this study, HbA1c levels showed a nonlinear, progressively accelerating relationship with urine ACR, suggesting that chronic hyperglycemia exerts cumulative and potentially exponential detrimental effects on renal function. Such patterns are indicative of the threshold-dependent exacerbation of oxidative stress, glycation end-product formation, and inflammatory mediators that collectively compromise glomerular integrity [21]. These observations are consistent with prior work, such as that by Bash et al., which linked poor glycemic control with increased inflammation and accelerated nephropathy progression even without albuminuria [22]. The positive associations between HbA1c and advanced proteinuria categories (e.g., 3+ and 4+) further emphasize the need for stringent glucose management to mitigate renal damage as projected by Arnold et al. [23].

Interestingly, the regression analyses revealed a nuanced relationship between SII, PLR, and urine protein categories. While SII showed consistently positive coefficients across most protein levels, PLR exhibited a shift towards negative coefficients at higher proteinuria categories. This divergence suggests potential threshold effects or nonlinear interactions that warrant further investigation. Such patterns might reflect adaptive immune responses or saturation effects, wherein the inflammatory pathways become less responsive or operate through different mechanisms at advanced stages of renal damage.

Overall, this study reinforces the role of systemic inflammation as a central mechanism in diabetic proteinuria and highlights the potential of SII and PLR as accessible, cost-effective biomarkers for assessing disease severity. Their correlations with ACR and urine protein levels support their integration into clinical practice for early detection and monitoring of diabetic nephropathy. Additionally, the association between HbA1c and proteinuria severity underscores the importance of rigorous glycemic control as a cornerstone of nephropathy prevention and management.

Limitations

The study’s retrospective and cross-sectional design precludes establishing causal relationships between systemic inflammatory markers (PLR and SII) and proteinuria in T2DM. The study was conducted at a single center with a relatively small sample size, potentially limiting the generalizability of findings to broader populations. Unmeasured confounding factors, such as dietary influences and medication adherence, may have impacted the observed associations. Also in this study, the medications for proteinuric and non-proteinuric patients, especially RAS-inhibitors or SGLT2-inhibitors, were not categorized in the table, despite their potential impact on SII or PLR. Future studies should consider stratifying patients based on medication use to better assess these effects.

## Conclusions

This study highlights the pivotal role of systemic inflammation in the progression of diabetic proteinuria and the clinical utility of SII and PLR as promising biomarkers for assessing disease severity and progression. The significant association between HbA1c and proteinuria severity underscores the importance of maintaining optimal glycemic control to mitigate renal damage and delay the onset of advanced DKD. Integrating SII and PLR into clinical practice, alongside traditional measures of glycemic control, could enhance the management and monitoring of DM-related renal complications. Future research should explore the longitudinal dynamics of these biomarkers and their responsiveness to therapeutic interventions, such as anti-inflammatory or glucose-lowering treatments. Investigating the molecular pathways underlying their association with renal dysfunction will further elucidate their role in the pathophysiology of diabetic complications. These efforts could pave the way for targeted therapies that address inflammation as a modifiable risk factor in DM-related kidney disease.

## References

[1] Hoogeveen EK (2022). The epidemiology of diabetic kidney disease. Kidney Dial.

[2] Fenta ET, Eshetu HB, Kebede N (2023). Prevalence and predictors of chronic kidney disease among type 2 diabetic patients worldwide, systematic review and meta-analysis. Diabetol Metab Syndr.

[3] Gupta S, Dominguez M, Golestaneh L (2023). Diabetic kidney disease: an update. Med Clin North Am.

[4] Alicic RZ, Rooney MT, Tuttle KR (2017). Diabetic kidney disease: challenges, progress, and possibilities. Clin J Am Soc Nephrol.

[5] Galicia-Garcia U, Benito-Vicente A, Jebari S (2020). Pathophysiology of type 2 diabetes mellitus. Int J Mol Sci.

[6] Guo W, Song Y, Sun Y (2022). Systemic immune-inflammation index is associated with diabetic kidney disease in Type 2 diabetes mellitus patients: Evidence from NHANES 2011-2018. Front Endocrinol (Lausanne).

[7] Li J, Wang X, Jia W (2024). Association of the systemic immuno-inflammation index, neutrophil-to-lymphocyte ratio, and platelet-to-lymphocyte ratio with diabetic microvascular complications. Front Endocrinol (Lausanne).

[8] Taslamacioglu Duman T, Ozkul FN, Balci B (2023). Could systemic inflammatory index predict diabetic kidney injury in type 2 diabetes mellitus?. Diagnostics (Basel).

[9] (2025). 2. Diagnosis and Classification of Diabetes: Standards of Care in Diabetes-2025. Diabetes Care.

[10] R: A language and environment for statistical computing, Vienna Vienna, Austria Austria (2024). The R Project for Statistical Computing. November 15, 2024.

[11] Donate-Correa J, Luis-Rodríguez D, Martín-Núñez E (2020). Inflammatory targets in diabetic nephropathy. J Clin Med.

[12] Li L, Chen K, Wen C, Ma X, Huang L (2024). Association between systemic immune-inflammation index and chronic kidney disease: a population-based study. PLoS One.

[13] Wu T, Ding L, Andoh V, Zhang J, Chen L (2023). The mechanism of hyperglycemia-induced renal cell injury in diabetic nephropathy disease: an update. Life (Basel).

[14] Yan P, Yang Y, Zhang X (2023). Association of systemic immune-inflammation index with diabetic kidney disease in patients with type 2 diabetes: a cross-sectional study in Chinese population. Front Endocrinol (Lausanne).

[15] Ye Z, Hu T, Wang J, Xiao R, Liao X, Liu M, Sun Z (2022). Systemic immune-inflammation index as a potential biomarker of cardiovascular diseases: a systematic review and meta-analysis. Front Cardiovasc Med.

[16] Aneez FA, Shariffdeen N, Haleem FA (2024). Correlation between neutrophil to lymphocyte ratio and platelet to lymphocyte ratio with proteinuria in different stages of chronic kidney disease. Egypt J Intern Med.

[17] Li P, Xia C, Liu P, Peng Z, Huang H, Wu J, He Z (2020). Neutrophil-to-lymphocyte ratio and platelet-to-lymphocyte ratio in evaluation of inflammation in non-dialysis patients with end-stage renal disease (ESRD). BMC Nephrol.

[18] Qin Z, Li H, Wang L, Geng J, Yang Q, Su B, Liao R (2022). Systemic immune-inflammation index is associated with increased urinary albumin excretion: a population-based study. Front Immunol.

[19] Trimarchi H, Muryan A, Dicugno M (2012). Proteinuria: an ignored marker of inflammation and cardiovascular disease in chronic hemodialysis. Int J Nephrol Renovasc Dis.

[20] Kumar M, Dev S, Khalid MU (2023). The bidirectional link between diabetes and kidney disease: mechanisms and management. Cureus.

[21] Amorim RG, Guedes GD, Vasconcelos SM, Santos JC (2019). Kidney disease in diabetes mellitus: cross-linking between hyperglycemia, redox imbalance and inflammation. Arq Bras Cardiol.

[22] Bash LD, Selvin E, Steffes M, Coresh J, Astor BC (2008). Poor glycemic control in diabetes and the risk of incident chronic kidney disease even in the absence of albuminuria and retinopathy: Atherosclerosis Risk in Communities (ARIC) Study. Arch Intern Med.

[23] Arnold F, Kappes J, Rottmann FA, Westermann L, Welte T (2024). HbA1c-dependent projection of long-term renal outcomes. J Intern Med.

